# Impact of difficult-to-treat depression for patients and society: a real-world study

**DOI:** 10.3389/fpsyt.2025.1702137

**Published:** 2025-12-09

**Authors:** Sergio Benavente, Alba Parra, Jorge Lopez-Castroman, Ismael Conejero, Pablo Alonso-Torres, Tatiana Caraballo López, Pablo Carrasco Arteaga, Enrique Baca-García

**Affiliations:** 1 Departamento de Psiquiatría, Hospital Universitario Infanta Elena Valdemoro, Madrid, Spain; 2 Department of Psychiatry, Nimes University Hospital, Nimes, France; 3 Institut de Génomique Fonctionnelle, University of Montpellier, Centre national de la recherche scientifique-L’Institut national de la santé et de la recherche médicale (CNRS-INSERM), Montpellier, France; 4 Centro de Investigación Biomédica en Red de Salud Mental (CIBERSAM), Research Group CB/07/09/0025, Madrid, Spain; 5 Department of Psychiatry, Radiology, Public Health, Nursing and Medicine, University of Santiago de Compostela, Santiago de Compostela, Spain; 6 Department of Psychiatry, Centre Hospitalier Universitaire (CHU) Nîmes, Neuropsychiatrie recherche épidémiologique et clinique (PSNREC), L’Institut national de la santé et de la recherche médicale (INSERM), University of Montpellier, Nîmes, France; 7 Department of Psychiatry, Instituto de Investigación Sanitaria Fundación Jiménez Díaz, Madrid, Spain; 8 Department of Psychiatry, Universidad Autónoma de Madrid, Madrid, Spain; 9 Department of Psychiatry, Hospital Universitario Fundación Jiménez Díaz, Madrid, Spain; 10 Market Access Department, Johnson & Johnson, Madrid, Spain; 11 Medical Department, Johnson & Johnson, Madrid, Spain; 12 Department of Psychiatry, Hospital Rey Juan Carlos, Móstoles, Madrid, Spain; 13 Universidad Católica del Maule, Talca, Chile; 14 Department of Psychiatry, Hospital Universitario General de Villalba, Madrid, Spain

**Keywords:** depression, major depressive disorder (MDD), treatment resistant depression (TRD), difficult-to-treat depression (DTD), suicide risk, costs

## Abstract

**Background:**

Treatments and evolution of depressive disorders are highly heterogeneous, with difficult-to-treat depression (DTD) presenting elevated medical and economic burdens, particularly when accompanied with suicidality. This study analyzed clinical profiles, evolution, and costs associated with major depressive disorder (MDD), MDD with suicide risk (MDD-SR), DTD, and DTD with suicide risk (DTD-SR) over 12 months, considering initial healthcare pathways.

**Methods:**

A cohort of 3,941 individuals aged ≥18 years was recruited between 2014 and 2018 in four Madrid hospitals. Patients were classified according to their first contact with mental health services through emergency settings (emergency-first) or outpatient settings (outpatient-first). Sociodemographic data, International Classification of Diseases (ICD-10) diagnoses, and healthcare resource use were extracted from electronic health records and the MeMind digital ecosystem. Suicide risk was assessed using the Mini International Neuropsychiatric Interview (MINI)-based suicide risk assessment, and clinical profiles and costs were compared.

**Results:**

Compared with outpatient-first patients, emergency-first patients showed greater depression severity, psychiatric comorbidities, and suicide risk (p < 0.001), along with increased rates of DTD (p = 0.021), poorer treatment outcomes, and higher global costs. Patients with DTD or suicide risk displayed greater depression severity, lower treatment response, more frequent relapses, psychiatric hospitalization, and antidepressant augmentation strategies compared to MDD-only patients. Emergency-first DTD-SR patients incurred the highest costs (€15,358.1 [SD = 16,415.1]/patient/year). Suicide risk was strongly associated with probable relapses and DTD.

**Conclusions:**

Despite the high economic burden, important needs remain unmet for DTD, especially for patients showing suicide risk with a first contact through the emergency setting. Earlier detection, innovative treatments, improved access to healthcare, and integration in mobile health programs should mitigate these gaps and improve clinical outcomes in this vulnerable population.

## Introduction

Depression is a common mental disorder (1), whose prevalence reaches 6.8% in Europe (2) and 4.7% in the Spanish population (3). It is one of the three largest causes of disability worldwide (4). While major depressive disorder (MDD) has often been considered a transient condition, epidemiological data suggest that above 30% of depressed individuals do not achieve remission following treatment (5, 6), despite augmentation pharmacotherapy (7). In addition, MDD is a risk factor for suicide, with a relative risk exceeding 7.6 compared to the general population (8), especially in individuals with treatment-resistant depression (9). While the European Medicines Agency and the US Food and Drug Administration defined treatment-resistant depression in patients as failing to achieve response to at least two adequate antidepressant trials (10), its definition differs between studies, as consensus is lacking on the operationalization of dose, duration, and response measures (11). Furthermore, it does not include other non-pharmacological interventions (12). The chronicity of symptoms may relate to the presence of comorbidities, poor treatment tolerance, care delays, and psychosocial stressors (5, 13). As a result, a pragmatic approach entailed the concept of difficult-to-treat depression (DTD) (6, 14). DTD refers to patients experiencing MDD with ongoing significant burden despite multiple treatments, including non-pharmacological interventions, and affects 15%–25% of patients with depression (12).

Studies in patient characteristics, disease burden, and patterns of clinical care for depression are scarce in Europe (15), and larger observational studies are warranted. Real-world data from electronic health records (EHRs) provide information about typical treatment interventions and naturalistic outcomes, thereby enriching the results obtained from controlled clinical trials (12, 15). Such digital-based research enables the optimization of clinical care in the real world for patients with DTD, identifies unmet needs, and guides large-scale health policies. This real-world naturalistic study aimed to describe and compare clinical characteristics, evolution, treatment patterns, healthcare resource utilization, and direct medical and indirect costs over a 12-month period, considering two subcohorts of depressed individuals entering mental healthcare through i) first contact with an emergency setting (emergency-first patients) or ii) first contact with an outpatient mental health service (outpatient-first patients). In both subcohorts, we assessed these outcomes according to depression subtypes (MDD or DTD) and suicide risk. Finally, we explored the clinical factors associated with suicide risk and the occurrence of DTD over the follow-up period.

## Materials and methods

### Study design and patient sample

We recruited depressed patients aged over 18 years who had a first contact with mental health services between 2014 and 2018 in four different hospitals belonging to the Department of Psychiatry of the Fundación Jiménez Díaz Hospital, Madrid, Spain.

Patients were included if they first contacted an emergency setting or an outpatient facility (initiating pharmacological or non-pharmacological antidepressant treatment), and if they were diagnosed with a depressive episode (F32.x) or recurrent depressive disorder (F33.x) according to the ICD-10 criteria. Patients with persistent mood disorder (F34.x), other depressive episodes (F32.8), or other recurrent (F33.8) or unspecified depressive disorders (F32.A) were not included in the study. The follow-up period extended over 12 months.

### Data and measures

Patients’ data were collected using the MeMind platform. MeMind is a digital ecosystem for behavioral monitoring using active ecological momentary assessments (16). The following information was drawn from EHRs: sociodemographic data, coded ICD-10 medical and psychiatric diagnoses, and data regarding healthcare resource use, including hospital admissions, visits to the emergency department, and attendance at psychiatry or psychology outpatient appointments. The Anatomical Therapeutic Chemical (ATC) classification was used to group the treatments as psychotropic, antidepressant, or non-psychotropic medications.

A combination of several coded indicators (compliance with outpatient follow-up, emergency visits, and hospital readmissions) were employed to identify the relapse of mental disorder according to the definition of Migoya-Borja et al. (17), which includes i) an inadequate administrative follow-up involving a failure to attend ≥1 psychiatric visit or an advance of ≥1 appointment within a 6-month period and ii) at least one visit to the emergency room for psychiatric reasons or a psychiatric hospitalization (17). According to Morrens et al. (18), patients were considered responders to treatment if they showed a decrease in Clinical Global Impression-Severity (CGI-S) score of ≥2 points from baseline or had a CGI-S score of ≤3 (18). Over the study period, including first contact with mental health services, suicide risk was identified if patients required referral to a suicide treatment program, visited emergency facilities due to suicidal ideation or attempts, or screened positive for suicide risk using the MINI scale. DTD was identified over the follow-up period in patients who showed problems in finding tolerable and effective treatment after the use of at least four pharmacological or non-pharmacological treatments, including at least two antidepressants (5, 12).

Consequently, patients were classified into four depression subtypes: MDD, major depressive disorder with suicide risk (MDD-SR), DTD, and difficult-to-treat depression with suicide risk (DTD-SR). Finally, costs over 1 year were calculated using the methodology of EPIdemiología y COstes en depresión (EPICO) (3).

EHRs were anonymized in compliance with Spanish laws on the Protection of Personal Data and the guarantee of digital rights. The authors assert that all procedures contributing to this work comply with the ethical standards of the relevant national and institutional committees on human experimentation and with the Declaration of Helsinki 1975, as revised in 2013. All procedures involving human subjects/patients were approved by the Fundación Jiménez Díaz Hospital Ethics Committee, and patients’ information was handled as stated in Spanish and European regulations on data protection and patients’ digital rights. All the participants provided written informed consent before entering the study and installing the MeMind application.

### Statistical analysis

We performed all statistical analyses using the Statistical Package for the Social Sciences (SPSS) version 29. First, we offered descriptive statistics of the whole population and performed univariate analyses to compare the clinical characteristics between i) the emergency-first patients and ii) the outpatient-first patients. In both subcohorts, we compared the evolution, the healthcare resource utilization, the treatment patterns, and costs between depression subtypes (MDD or DTD) and according to associated suicide risk (MDD-SR and DTD-SR). We performed the univariate analyses using Fisher’s exact test, chi-square, or ANOVA. We conducted a Kaplan–Meier survival analysis to estimate the occurrence of mental disorder relapse over the 12-month follow-up period. Additionally, we constructed a multivariate Cox regression model to explore the factors associated with such events. For exploratory purposes, we performed a binary logistic regression to assess the clinical factors associated with suicide risk, the occurrence of DTD, and the response to treatment. The significance level was set at p < 0.05, using two-sided tests and 95% confidence intervals.

## Results

### Clinical characteristics of the whole sample

**Figure 1:**
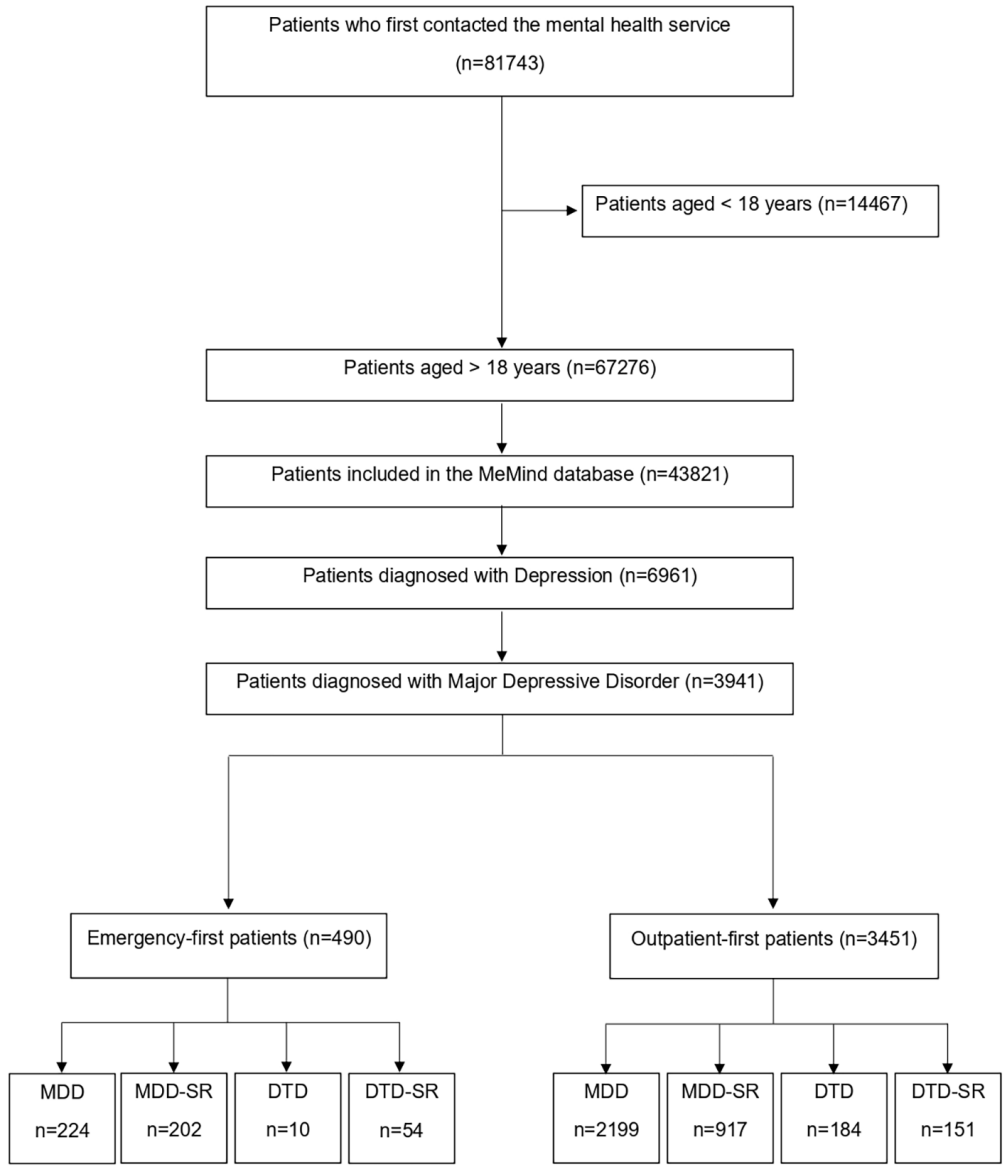
Study flowchart. MDD, major depressive disorder; MDD-SR, major depressive disorder with suicide risk; DTD, difficult-to-treat depression; DTD-SR, difficult-to-treat depression with suicide risk.

Among the 81,743 patients who received initial mental healthcare, 67,276 were 18 years old or above. Of these, 43,821 authorized their inclusion in the MeMind program. Within this group, 6,961 patients showed depression, and 3,941 were diagnosed with a major depressive episode and were included in the analyses (**Figure 1**).

Patient clinical characteristics at inclusion are reported in **Table 1**. Sample mean age was 50.4 [SD = 16.8], and 67.1% of the patients were women (N = 2,644). Eighty percent of the patient sample was of working age, but only 45.4% were currently employed, and 18% reported a work disability (temporary or permanent). More specifically, permanent or temporary work disability was observed in 3.30% and 11.30% of the MDD population, 5.30% and 14.30% of the MDD-SR population, 9.90% and 19.90% of the DTD population, and 8.60% and 28.40% of the DTD-SR population, respectively. Suicide risk affected 33.6% (N = 1,324) of the patients, and over the 12 months of follow-up, DTD was identified in 399 individuals (10.1%). Non-response to treatment affected 66.7% (N = 630) of patients showing MDD-SR, 65% (N = 115) of those with DTD, 82.2% (N = 162) of patients showing DTD-SR, and 44.3% (N = 834) of those with MDD (p < 0.001). Within the whole sample, 5.4% of individuals showed a probable relapse of mental disorder. More specifically, a probable relapse affected 8.4% of MDD-SR patients (N = 94/1,119), 5.7% of DTD patients (N = 11/194), 24.4% of DTD-SR patients (N = 50/205), and 2.4% of MDD patients (N = 57/2,423) (p < 0.001). Compared with MDD patients, the risk of relapse was three times as high in the MDD-SR subgroup (HR 3.012; 95% CI, 2.167–4.187; p < 0.001) and more than six times higher in the DTD-SR subgroup (HR 6.685; 95% CI, 4.570–9.779; p < 0.001).

**Table 1:** Clinical characteristics of depressed individuals according to the first contact with mental health services ^a^.

Clinical characteristics	Whole sample (n = 3,941)	First contact with mental health service		Significance (p-value)^b^
		Emergency-first patients (n = 490)	Outpatient-first patients (n = 3,451)	
Age [SD]	50.4 [16.8]	48.1 [51.6]	50.8 [16.8]	0.001
Gender (female)	2,644 (67.1%)	280 (57.1%)	2,364 (68.5%)	0.002
First CGI-S measure				<0.001
Not evaluated	458 (11.6%)	77 (15.7%)	381 (11%)	
Borderline mentally ill	280 (7.1%)	4 (0.8%)	276 (8%)	
Mildly ill	1,094 (27.8%)	98 (20%)	996 (28.9%)	
Moderately ill	1,786 (45.3%)	231 (47.1%)	1,555 (45.1%)	
Markedly/severely ill	323 (8.2%)	80 (16.3%)	243 (7%)	
Comorbidities				
Substance use disorders	237 (6%)	43 (8.8%)	194 (5.6%)	0.008
Personality disorders	306 (7.8%)	55 (11.2%)	251 (7.3%)	0.004
Somatic illness	580 (14.7%)	39 (8%)	541 (15.7%)	<0.001
Depression subtypes				
Global occurrence of SR	1,324 (33.6%)	256 (52.2%)	1,068 (30.9%)	<0.001
Global occurrence of DTD	399 (10.1%)	64 (13.1%)	335 (9.7%)	0.021
MDD	2,423 (61.5%)	224 (45.7%)	2,199 (63.7%)	<0.001
MDD-SR	1,119 (28.4%)	202 (41.2%)	917 (26.6%)	
DTD	194 (4.9%)	10 (2%)	184 (5.3%)	
DTD-SR	205 (5.2%)	54 (11%)	151 (4.4%)	
Treatment outcomes				
Inadequate administrative follow-up	1,750 (44.4%)	200 (41%)	1,550 (45%)	0.089
Non-response to treatment ^c^	1,741 (54.4%)	219 (62.2%)	1,522 (53.4%)	0.002
Probable relapse of mental disorder	212 (5.4%)	108 (22%)	104 (3%)	<0.001
>75% attendance at psychiatry consultation	2,526 (64.1%)	242 (49.4%)	2,284 (66.2%)	<0.001
>75% attendance at psychology consultation	900 (22.8%)	78 (15.9%)	822 (23.8%)	0.273
Healthcare resource use				
Non-psychiatric hospitalization	687 (17.4%)	272 (55.5%)	415 (12%)	<0.001
Non-psychiatric emergency consultation	1,986 (50.4%)	489 (99.8%)	1,497 (43.4%)	<0.001
Psychiatric hospitalization	329 (8.3%)	253 (51.6%)	76 (2.2%)	<0.001
Psychiatric emergency consultation	573 (14.5%)	490 (100%)	83 (2.4%)	<0.001
Yearly direct medical and indirect mean costs (€/patient/year)^d^				
**Psychiatric direct medical costs**	1,212.3 [3,277.3]	4,457.0 [7,476.2]	752.4 [1,627.9]	<0.001
Use of mental healthcare resources	831.9 [3,007.3]	3,863.8 [7,019.5]	402.2 [1,366.1]	<0.001
Psychiatric medication	380.4 [676.7]	593.2 [956.3]	350.2 [621.3]	<0.001
**Somatic direct medical costs**	1,353.9 [2,388.5]	1,337.7 [2,190.8]	1,356.1 [2,415.5]	0.436
Use of general healthcare resources	1,253.3 [2,348.3]	1,222.2 [2,136.7]	1,257.7 [2,377.1]	0.023
Medication for somatic disorders	100.6 [324.1]	115.5 [349.2]	98.4 [320.3]	<0.001
**Global costs**	4,475.2 [8,108.2]	7,386.5 [11,265.5]	4,062.5 [7,465.3]	<0.001
Use of mental and general healthcare resources	2,085.2 [3,860.9]	5,086.0 [7,461.8]	1,659.9 [2,771.8]	<0.001
Psychiatric and somatic medication	481.0 [835.1]	708.7 [1,125.9]	448.6 [779.9]	<0.001
Temporary work disability	815.6 [4,140.5]	749.6 [3,983.8]	824.9 [4,162.7]	0.706
Permanent work disability	1,093.4 [5,203.4]	842.2 [4,633.2]	1,129.1 [5,279.1]	0.208

MDD, major depressive disorder; SR, suicide risk; MDD-SR, major depressive disorder with suicide risk; DTD, difficult-to-treat depression; DTD-SR, difficult-to-treat depression with suicide risk.

a Data are means [standard deviation] or number (%).

b The characteristics of the patients who first contacted the emergency setting were compared with the characteristics of those who first contacted the outpatient mental health service.

c The percentage of non-responders has been calculated over the number of patients with available CGI measurement.

d Use of mental healthcare resources: psychiatric admissions, psychiatric emergencies, psychiatric consultation and psychotherapy, neurostimulation and neuromodulation treatments; psychiatric medications: antidepressants, anxiolytics, and antipsychotics; use of general healthcare resources: non-psychiatric admissions, non-psychiatric emergencies, non-psychiatric consultations, lab studies, and imaging for somatic disorders. Indirect costs due to temporary and permanent work disability were included in global cost calculations.

Patients diagnosed with DTD and DTD-SR were more frequently prescribed augmentation strategies with antipsychotics or mood stabilizers during the follow-up (74.2%, N = 144; and 83.9%, N = 172, respectively). The augmentation strategy was the most used in 26.7% (N = 645) of MDD patients and in 34.8% (N = 388) of MDD-SR patients.

Finally, the 1-year global costs per patient averaged 4,475.2 [SD = 8,108.2] euros in the whole sample (**Table 1**).

### Initial healthcare pathway and clinical profiles

Emergency-first patients were less frequently women than outpatient-first patients (p = 0.002); they were also younger (p = 0.001) and more likely to present a complicated depression [DTD and/or suicide risk (SR)] (p < 0.001). Rates of global suicide risk and global DTD were higher in emergency-first patients than in outpatient-first patients (p < 0.001 and p = 0.021, respectively). The detailed results for depression subtypes are shown in **Table 1**.

The patterns of comorbidity, treatment outcomes, and healthcare resource use were different between the subcohorts: substance use disorder and personality disorder were more frequent in emergency-first patients compared to outpatient-first patients (p = 0.008 and p = 0.004, respectively), whereas rates of somatic illnesses were higher in outpatient-first patients (p < 0.001).

**Figure 2:**
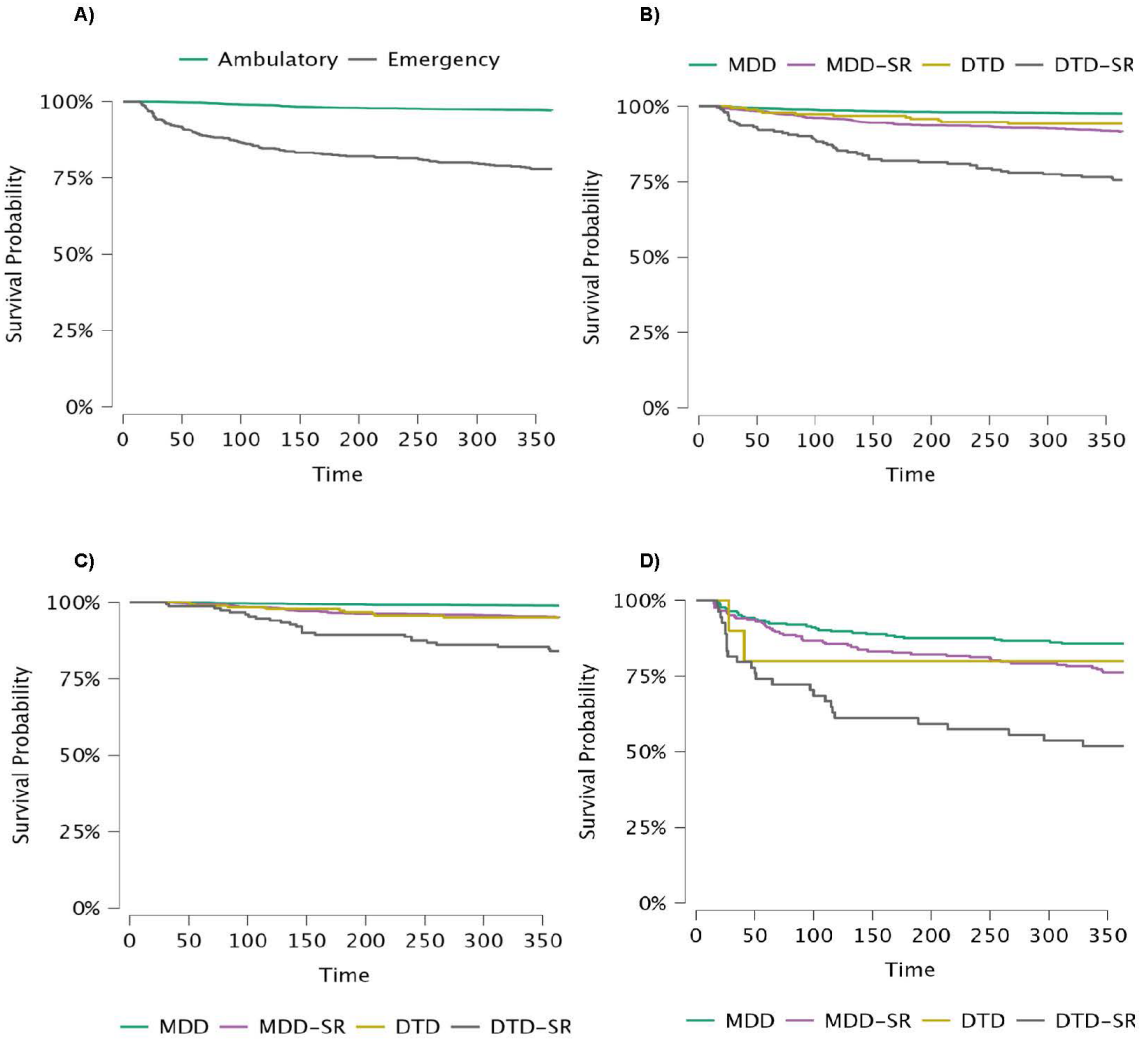
Relapse rate during the 1-year follow-up period. **(A)** Whole sample (n = 3,941) according to the first contact with mental healthcare resources (Log Rank 501.472; gl = 2; p < 0.001). **(B)** Whole sample (n = 3,941) according to the depression subtypes (Log Rank 122.182; gl = 3; p < 0.001). **(C)** Outpatient-first patient subgroup (n = 3,451) according to depression subtypes (Log Rank 72.033; gl = 3; p < 0.001). **(D)** Emergency-first patient subgroup (n = 490) according to depression subtypes (Log Rank 5.929; gl = 3; p < 0.115).

Compared to outpatient-first patients, those who first attended the emergency setting exhibited the following: i) Higher non-response rates (p = 0.002), ii) higher rates of antidepressant augmentation as most frequently used strategy at 54.7% (n = 262) vs. 31.5% (n = 1,087) (<0.001), iii) more frequent probable relapse (**Figure 2**; HR 3.393; 95% CI, 2.964–3.885; p < 0.001), and iv) greater use of healthcare resources, including psychiatric emergency consultation or psychiatric hospitalization (p < 0.001 and p < 0.001, respectively).

Finally, the 1-year mean global costs per patient were higher in emergency-first patients than in outpatient-first patients subcohort (p < 0.001; **Table 1**).

### Depression subtypes and clinical outcomes in outpatient-first patients

**Figure 3:**
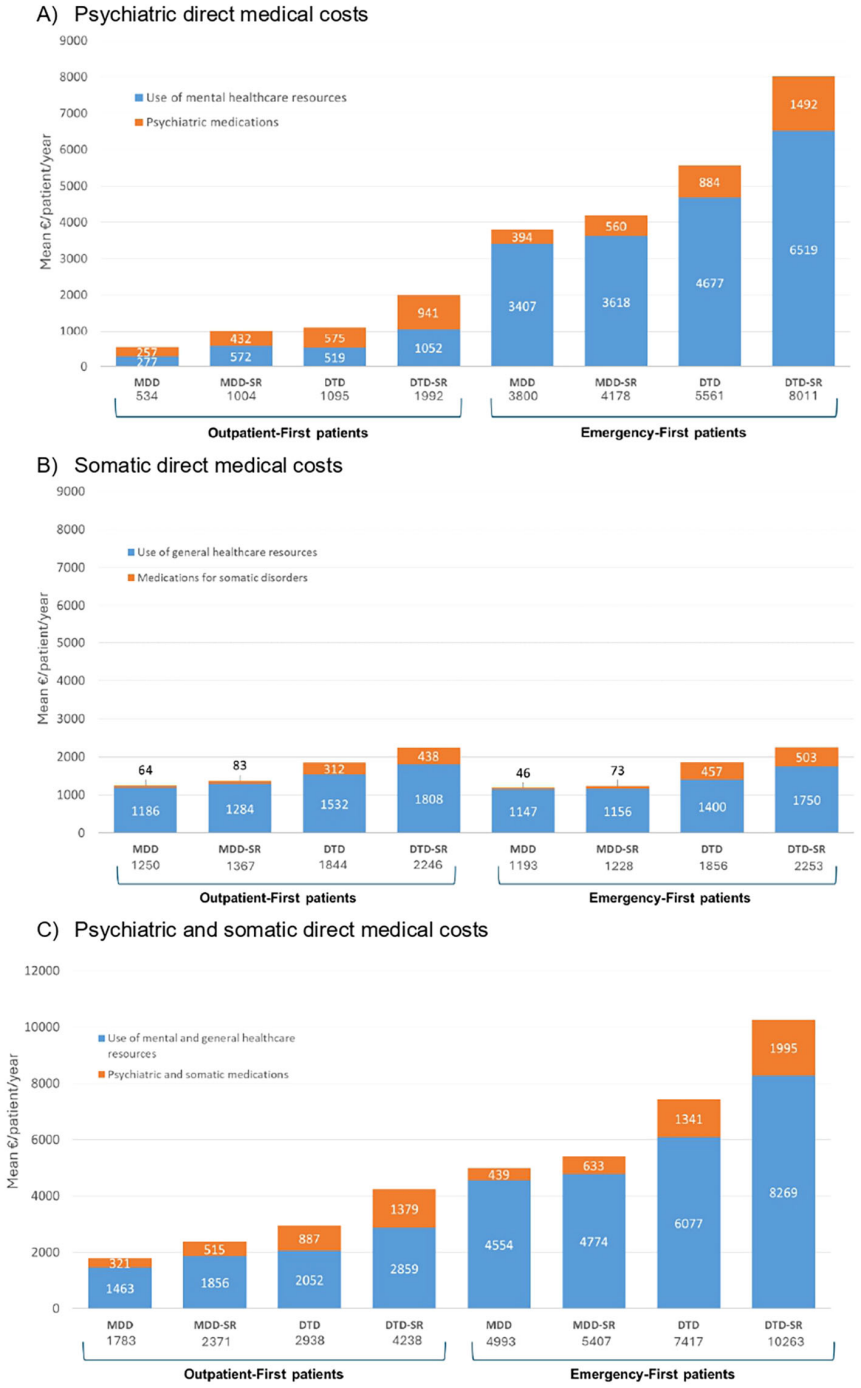
Cumulative effect of initial healthcare pathway and depressive phenotype on direct medical costs measured at 1-year follow-up. National Health System (NHS) perspective. **(A)** Psychiatric direct medical costs. **(B)** Somatic direct medical costs. **(C)** Psychiatric and somatic direct medical costs.

**Figure 4:**
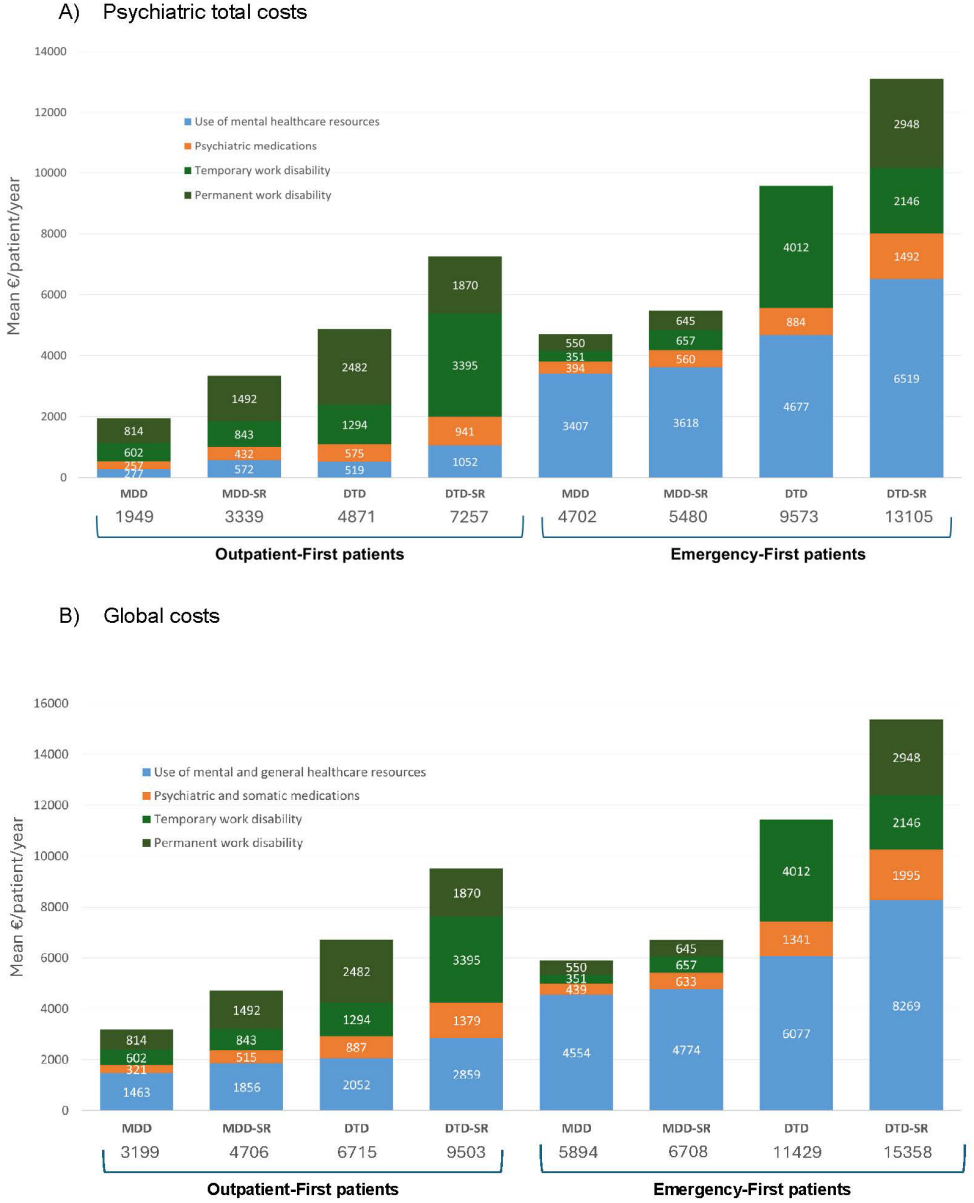
Cumulative effect of initial healthcare pathway and depressive phenotype on psychiatric total costs and global costs measured at 1-year follow-up. Societal perspective. **(A)** Psychiatric total costs. **(B)** Global costs.

The patients with DTD-SR showed the highest clinical severity (rates of markedly/severely ill 19.2%; p < 0.001) and the highest rates of work disability (temporary or permanent, 37.6%), non-response to treatment (81.2%), probable relapse of mental disorder (16%), and psychiatric hospitalizations (9.9%) and required most frequently antidepressant augmentation strategies (80.8%; **Supplementary Table 1**). Mean psychiatric and somatic direct medical costs per patient over 1 year were higher in the patients with DTD-SR than in all the other subgroups (**Figure 3**), as well as mean global cost per patient over 1 year (**Figure 4**; DTD-SR, 9,503.1 [SD = 10,561.4] euros; MDD, 3,198.8 [SD = 6,379.0] euros; MDD-SR, 4,705.9 [SD = 8,156.7] euros; DTD, 6,714.8 [SD = 9,745.3] euros; p < 0.001).

### Depression subtypes and clinical outcomes in emergency-first patients

The patients with DTD-SR showed the highest clinical severity (rates of markedly/severely ill 22.2%; p < 0.001), the highest rates of work disability (temporary or permanent, 35.3%), the highest rates of non-response to treatment (85.4%), the highest rates of probable relapse of mental disorder (48%), and the highest rates of psychiatric hospitalizations (72.2%; **Supplementary Table 2**). Mean psychiatric and somatic direct medical costs per patient over 1 year (**Figure 3**) and global costs per patient over 1 year (**Figure 4**) were also higher in the patients with DTD-SR (15,358.1 [SD = 16,415.1] euros) than in all the other subgroups (MDD, 5,894.1 [SD = 9,811.3]; MDD-SR, 6,708.1 [SD = 10,135.9]; DTD, 11,429.2 [SD = 12,710.1] euros, p < 0.001).

### Suicide risk, DTD, and response to treatment in the whole sample

We built two forward likelihood ratio logistic regression models. The risk factors influencing DTD were suicide risk (OR = 1.839; 95% CI, 1.466–2.307), permanent work disability with respect to active employment (OR = 2.193; 95% CI, 1.416–3.396), temporary work disability with respect to active employment (OR = 2.129; 95% CI, 1.587–2.857), comorbid somatic disorder (OR = 1.551; CI–95% 1.172–2.053), comorbid mental disorder (OR = 1.777; 95% CI, 1.404–2.250), probable relapse of mental disorder (OR = 2.570; 95% CI, 1.805–3.659), and female gender (OR = 1.524; 95% CI, 1.193–1.948).

The factors associated with suicide risk were DTD (OR = 1.567; 95% CI, 1.237–1.984), temporary work disability with respect to active employment (OR = 1.266; 95% CI, 1.0211.569), comorbid mental disorder (OR = 1.446; 95% CI, 1.227–1.704), probable relapse of mental disorder (OR = 3.092; 95% CI, 2.257–4.237), poor compliance with programmed healthcare consultations (OR = 1.251; 95% CI, 1.083–1.445), birth outside Spain (OR = 1.306; 95% CI, 1.062–1.607), and being unemployed with respect to active employment (OR = 1.393; 95% CI, 1.098–1.768).

Finally, the factors associated with the response to treatment in logistic regression were as follows: being free from DTD (OR = 1.708; 95% CI, 1.294–2.253), being free from suicide risk (OR = 2.184; 95% CI, 1.844–2.587), compliance with scheduled appointments (OR = 1.524; 95% CI, 1.294-1.795), being free from comorbid somatic illness (OR = 1.336; 95% CI, 1.079–1.655), no intake of antipsychotics (OR = 1.753; 95% CI, 1.443–2.129), and receiving psychotherapy (OR = 1.314; 95% CI, 1.105–1.562).

## Discussion

### Main findings

In this naturalistic real-world study, we investigated the severity of depression, its complications and outcomes, the use of healthcare resources over the follow-up period, and its economic impact according to the initial care pathways of 3,941 patients diagnosed with MDD. Emergency-first patients showed higher depression severity, higher presence of psychiatric comorbidities, higher suicide risk, higher rates of DTD, poorer treatment outcomes, greater use of healthcare resources, and higher global costs than outpatient-first patients. The patients with DTD or showing suicide risk had higher depression severity, lower response-to-treatment rates, higher rates of probable relapse, required psychiatric hospitalization, and antidepressant augmentation strategies more frequently than those with MDD only, whatever the initial care pathway. The mean psychiatric direct medical costs and mean global costs per patient over 1 year were the highest in the DTD-SR emergency-first subgroup. Finally, the occurrence of suicide risk and DTD shared common risk factors, including the existence of a temporary work disability, comorbid psychiatric disorders, and the probable relapse of mental disorder. Our longitudinal study involved one of the largest patient samples to date, reaching the population size (N = 3,671) described in the milestone naturalistic study conducted by Rush et al. (13). We highlighted several factors potentially associated with those outcomes and described their links with DTD and suicide risk, as Rush and colleagues have recently recalled that two-thirds of depressed individuals reached remission after four lines of treatment (19). Entering the clinical care pathway through initial admission to the emergency unit seems related to higher disorder severity and greater treatment complexity over the follow-up period. These results may outline increased psychiatric instability in this depressed patient subgroup. In a past non-comparative study, up to 9% and 13% of individuals may repeat self-harm or may be admitted to a psychiatric hospital within 30 days following a visit to the psychiatry emergency room (20), especially when associated with comorbid personality disorder. In our study, we found higher relapse rates (22%), as the definition adopted was broader.

Furthermore, the emergency subcohort showed far more likelihood of requiring psychiatric hospitalization over the follow-up period than the outpatient subcohort. One may hypothesize that starting the treatment of complex depressive disorders in an emergency psychiatric setting may also increase the risk of poor outcome, as such units are not resourced to initiate and organize long-term structured and multidimensional healthcare pathways, as was recently suggested (21).

The presence of both DTD and suicide risk led to more intensive psychiatric care, including psychiatric hospitalization, antidepressant augmentation strategies, and increased costs. As DTD is a recent concept, few studies have assessed the profiles of the patients affected. In our sample, the diagnosis of DTD was two times lower than the prevalence found in a study conducted in the United Kingdom (12), although the diagnostic criteria were all based on those of McAllister-Williams (22, 23). Although different data collection methods were applied, similar clinical patient profiles were obtained (12).

A recent European study reported baseline characteristics of DTD patients involved in a vagus nerve stimulation trial (24), illustrating the severity of the potential burden associated with DTD and the elevated frequency of relapses exceeding those found in treatment-resistant depression registries.

When combined with current suicide risk, the rates of non-treatment response, relapses, and non-psychiatric and psychiatric hospitalizations dramatically increased in patients with DTD. Overall, we found that non-response rates over 1 year exceeded those reported in patients with treatment-resistant depression in Europe (15). Furthermore, current suicide risk or suicidality may be considered an additional comorbidity, predicting by itself psychiatric hospitalization and independently reflecting the severity of psychiatric disorders, as shown (25), hence impairing further treatment outcomes in depressed individuals (26).

The clinical complexity of DTD, when combined with suicide risk in individuals entering a healthcare pathway through a visit to the emergency department, translates into increasing psychiatric direct medical costs and global costs. Prior Spanish real-world studies evaluated those medical costs in retrospective observational studies involving depressed people and showed that they ranged between a mean of €3,846/patient/year for non-treatment-resistant depression (non-TRD) patients and €6,096/patient/year in patients affected by a treatment-resistant depressive disorder (27). Our observations outline that, despite the high economic burden, important needs remain unmet in the DTD-SR patients, leading to frequent relapses and impaired outcomes.

To fill them, important efforts should be pursued for developing innovative treatment strategies and for rethinking initial care pathways, in particular from admission to the psychiatric emergency room. In parallel to increasing access to innovative treatment options, which were proven to be quickly effective (10), such as intranasal esketamine for treating TRD, or for the rapid reduction of depressive symptoms in MDD patients who, according to clinical judgment, are experiencing a psychiatric emergency situation such as suicidal ideation (28), emergency settings should connect their routine practices to long-term healthcare goals, for instance, by involving patients in integrated digital follow-up at the first visit (29). Also, the Canadian Network for Mood and Anxiety Treatments (CANMAT) recently updated its recommendations for treating DTD and proposed pharmacogenetic testing for the proper selection of adjunctive antidepressants (30).

### Limitations of the study

This study presents several limitations that should be considered when interpreting the findings. First, the operationalization of DTD, adapted from Costa et al (12)., may limit comparability with studies using more stringent definitions. The choice of DTD was motivated by its broader and more pragmatic scope, encompassing non-pharmacological interventions. Nonetheless, DTD does not completely resolve TRD’s definitional constraints. In our design, DTD was determined exclusively during follow-up, as this classification requires documented evidence of multiple prior treatment failures. Conversely, SR was assessed both at baseline and throughout the 12−month follow-up to capture persistent as well as newly emerging risk, given its clinical relevance in both contexts. However, this could make data interpretation more challenging. Second, the identification of probable relapse relied on administrative criteria described in prior literature (17), without incorporating patient-reported symptom data. While suitable to describe poor evolution in real−world evidence frameworks, this approach may underestimate subthreshold symptom fluctuations and does not allow the calculation of relapse rates exclusively among responders and remitters. Third, although emergency department presentations were analyzed as part of the initial care pathway, our dataset did not systematically record the specific reason for each presentation beyond diagnostic codes. This limits our ability to distinguish acute psychiatric situations from visits that may have been managed in outpatient settings. Fourth, the representativeness of the sample may be constrained by the requirement to participate in the MeMind digital monitoring program, potentially excluding individuals without smartphone access or sufficient digital literacy. Moreover, recruitment from four hospitals in Madrid may affect generalizability to other regions or healthcare systems. Fifth, differences between emergency−first and outpatient−first pathways may be influenced by unmeasured confounders such as socioeconomic status or health−seeking behavior. Cultural stigma could also reduce the accuracy of suicide risk assessments. Sixth, while the EPICO methodology used for cost calculation facilitates comparability within Spain, differences in cost components and valuation methods compared to other studies hinder direct cross−national comparisons. Finally, the retrospective observational design inherent to our approach also precludes causal inference, and residual confounding cannot be excluded.

## Conclusions

Overall, we reported the clinical profiles and the economic impact of MDD in patients differing in their initial healthcare pathway, with characterized suicide risk, and categorized them into new operational depression subtypes. We highlighted the treatment gap and unmet needs in individuals affected by DTD, who, despite the high economic expenses, suffer from frequent relapses and remain at risk of suicide. Further access and use of innovative treatments and integrated care programs should help to overcome these gaps, and those patients should be diagnosed early to benefit from secondary and tertiary prevention. To this end, the generalization of mobile health monitoring programs is a promising avenue.

## Data Availability

The original contributions presented in the study are included in the article/**Supplementary Material**. Further inquiries can be directed to the corresponding author.
