# Correction: Impact of difficult-to-treat depression for patients and society: a real-world study

**DOI:** 10.3389/fpsyt.2025.1770603

**Published:** 2026-01-08

**Authors:** Sergio Benavente, Alba Parra, Jorge Lopez-Castroman, Ismael Conejero, Pablo Alonso-Torres, Tatiana Caraballo López, Pablo Carrasco Arteaga, Enrique Baca-García

**Affiliations:** 1 Departamento de Psiquiatría, Hospital Universitario Infanta Elena Valdemoro, Madrid, Spain; 2 Department of Psychiatry, Nimes University Hospital, Nimes, France; 3 Institut de Génomique Fonctionnelle, University of Montpellier, Centre national de la recherche scientifique-L’Institut national de la santé et de la recherche médicale (CNRS-INSERM), Montpellier, France; 4 Centro de Investigación Biomédica en Red de Salud Mental (CIBERSAM), Research Group CB/07/09/0025, Madrid, Spain; 5 Department of Psychiatry, Radiology, Public Health, Nursing and Medicine, University of Santiago de Compostela, Santiago de Compostela, Spain; 6 Department of Psychiatry, Centre Hospitalier Universitaire (CHU) Nîmes, Neuropsychiatrie recherche épidémiologique et clinique (PSNREC), L’Institut national de la santé et de la recherche médicale (INSERM), University of Montpellier, Nîmes, France; 7 Department of Psychiatry, Instituto de Investigación Sanitaria Fundación Jiménez Díaz, Madrid, Spain; 8 Department of Psychiatry, Universidad Autónoma de Madrid, Madrid, Spain; 9 Department of Psychiatry, Hospital Universitario Fundación Jiménez Díaz, Madrid, Spain; 10 Market Access Department, Johnson & Johnson, Madrid, Spain; 11 Medical Department, Johnson & Johnson, Madrid, Spain; 12 Department of Psychiatry, Hospital Rey Juan Carlos, Móstoles, Madrid, Spain; 13 Universidad Católica del Maule, Talca, Chile; 14 Department of Psychiatry, Hospital Universitario General de Villalba, Madrid, Spain

**Keywords:** depression, major depressive disorder (MDD), treatment resistant depression (TRD), difficult-to-treat depression (DTD), suicide risk, costs

Author Pablo Alonso-Torres was erroneously assigned to affiliation ^9^Department of Psychiatry, Hospital Universitario Fundación Jiménez Díaz, Madrid, Spain. This affiliation has now been removed for author Pablo Alonso-Torres.

Author Tatiana Caraballo López was erroneously assigned to affiliation ^10^Market Access Department, Johnson & Johnson, Madrid, Spain. This affiliation has now been removed for author Tatiana Caraballo López.

Author Pablo Carrasco Arteaga was erroneously assigned to affiliation ^9^Department of Psychiatry, Hospital Universitario Fundación Jiménez Díaz, Madrid, Spain. This affiliation has now been removed for author Pablo Carrasco Arteaga.

Author Enrique Baca-Garcia was erroneously assigned to affiliation ^11^Medical Department, Johnson & Johnson, Madrid, Spain. This affiliation has now been removed for author Enrique Baca-Garcia.

Affiliation ^10^Market Access Department, Johnson & Johnson, Madrid, Spain was omitted for author Pablo Alonso-Torres. This affiliation has now been added for author Pablo Alonso-Torres.

Affiliation ^11^Medical Department, Johnson & Johnson, Madrid, Spain was omitted for author Tatiana Caraballo López. This affiliation has now been added for author Tatiana Caraballo López.

Affiliation ^10^Market Access Department, Johnson & Johnson, Madrid, Spain was omitted for author Pablo Carrasco Arteaga. This affiliation has now been added for author Pablo Carrasco Arteaga.

Affiliation ^9^Department of Psychiatry, Hospital Universitario Fundación Jiménez Díaz, Madrid, Spain was omitted from the paper. This affiliation has now been added for author Ismael Conejero.

The original article has been updated.

