# Supplementary material for: Impact of difficult-to-treat depression for patients and society: a real-world study
Source: Front Psychiatry. 2025 Dec 9;16:1702137. doi: 10.3389/fpsyt.2025.1702137 (PMC12724535; doi:10.3389/fpsyt.2025.1702137)
Supplement: Supplementary file 1 [file Table1.docx]

**Supplemental eTable 1. Clinical characteristics of outpatient-first patients according to the depression profile and complications.^a^**

| Clinical characteristics | Major Depressive Disorder (n= 2199) | Major Depressive Disorder with Suicide Risk  (n= 917) | Difficult to Treat Depression  (n=184) | Difficult to Treat Depression with Suicide Risk  (n=151) | Significance  (p-value)^b^ |
| --- | --- | --- | --- | --- | --- |
| **First CGI-S measure** |  |  |  |  | <0.001 |
| Not evaluated | 265 (12.1%) | 82 (8.9%) | 28 (15.2%) | 6 (4%) |  |
| Borderline mentally ill | 215 (9.8%) | 49 (5.3%) | 8 (4.3%) | 4 (2.6%) |  |
| Mildly ill | 780 (35.5%) | 158 (17.2%) | 40 (21.7%) | 18 (11.9%) |  |
| Moderately ill | 862 (39.2%) | 507 (55.3%) | 92 (50%) | 94 (62.3%) |  |
| Markedly/ Severely ill | 77 (3.5%) | 121 (13.2%) | 16 (8.7%) | 29 (19.2%) |  |
| **Treatment outcomes** |  |  |  |  |  |
| Inadequate administrative follow-up | 893 (41%) | 487 (53%) | 86 (47%) | 84 (56%) | <0.001 |
| Non-response to treatment^c^ | 761 (43.6%) | 532 (67.8%) | 108 (64.3%) | 121 (81.2%) | <0.001 |
| Probable relapse of mental disorder | 25 (1.1%) | 46 (5%) | 9 (4.9%) | 24 (16%) | <0.001 |
| > 75% attendance to Psychiatry consultation | 1459 (66.3%) | 558 (60.9%) | 150 (81.5%) | 117 (77.5%) | 0.001 |
| > 75% attendance to Psychology consultation | 447 (20.3%) | 238 (26%) | 70 (38%) | 67 (44.4%) | 0.007 |
| **Healthcare resource use** |  |  |  |  |  |
| Non-psychiatric Hospitalization | 229 (10.4%) | 129 (14.1%) | 27 (14.7%) | 30 (19.9%) | <0.001 |
| Non-psychiatric Emergency consultation | 881 (40.1%) | 432 (47.1%) | 89 (48.4%) | 95 (62.9%) | <0.001 |
| Psychiatric Hospitalization | 13 (0.6%) | 37 (4%) | 11 (6%) | 15 (9.9%) | <0.001 |
| Psychiatric Emergency consultation | 30 (1.4%) | 30 (3.3%) | 8 (4.3%) | 15 (9.9%) | <0.001 |
| **Most used antidepressant treatment strategy during the follow-up** |  |  |  |  | <0.001 |
| Psychotherapy and other non-antidepressant drug | 241 (11%) | 86 (9.4%) | 1 (0.5%) | 0 (0%) |  |
| Antidepressant monotherapy | 824 (37.5%) | 274 (29.9%) | 14 (7.6%) | 5 (3.3%) |  |
| Combination of antidepressants | 594 (27%) | 266 (29%) | 35 (19%) | 24 (15.9%) |  |
| Antidepressant Augmentation (antipsychotic or mood stabilizer) | 540 (24.6%) | 291 (31.7%) | 134 (72.8%) | 122 (80.8%) |  |
| Mean number of antidepressants | 1.6 (1.0) | 1.8 (1.2) | 2.5 (1.1) | 3.1 (1.3) | <0.001 |
| **Yearly direct medical and indirect mean costs (€/patient/year)^d^** |  |  |  |  |  |
| **Psychiatric direct medical costs** | 533.8 [982.3] | 1003.7 [2346.9] | 1094.7 [1906.7] | 1992.4 [2446.1] | <.001 |
| Use of mental healthcare resources | 277.0 [765.5] | 572.0 [2067.1] | 519.4 [1647.5] | 1051.7 [2025.6] | <.001 |
| Psychiatric medications | 256.8 [494.2] | 431.7 [704.4] | 575.3 [852.3] | 940.7 [895.4] | <.001 |
| **Somatic direct medical costs** | 1249.6 [2217.9] | 1367.3 [2673.4] | 1843.7 [2447.4] | 2245.8 [3147.7] | <.001 |
| Use of general healthcare resources | 1185.9 [2192.2] | 1284.2 [2652.5] | 1532.2 [2451.4] | 1807.6 [2961.5] | .023 |
| Medications for somatic disorders | 63.7 [237.3] | 83.1 [271.5] | 311.5 [529.4] | 438.2 [743.4] | <.001 |
| **Global costs** | 3198.8 [6379.0] | 4705.9 [8156.7] | 6714.8 [9745.3] | 9503.1 [10561.4] | <.001 |
| Use of mental and general healthcare resources | 1462.9 [2348.1] | 1856.2[3362.2] | 2051.6 [3028.3] | 2859.3 [3644.1] | <.001 |
| Psychiatric and somatic medications | 320.5 [590.9] | 514.8 [828.9] | 886.8 [1141.4] | 1378.9 [1309.3] | <.001 |
| Temporary work disability | 601.6 [3561.5] | 843.1 [4158.8] | 1294.2 [5265.6] | 3394.7 [8098.8] | <.001 |
| Permanent work disability | 813.8 [4470.0] | 1491.8 [6142.2] | 2482.2 [7411.8] | 1870.2 [6762.7] | <.001 |

^a^Data are means [Standard Deviation], or number (%).

^b^The characteristics of the patients who contacted first the outpatient mental health service were compared according to the depression profile.

^c^The percentage of non-responders has been calculated over the N of patients with available CGI measurement.

^d^Use of mental healthcare resources: psychiatric admissions, psychiatric emergencies, psychiatric consultation and psychotherapy, neurostimulation and neuromodulation treatments; psychiatric medications: antidepressant, anxiolytics and antipsychotics; use of general healthcare resources: non-psychiatric admissions, non-psychiatric emergencies, non-psychiatric consultations, lab studies and imaging for somatic disorders. Indirect costs due to temporary and permanent work disability were included in global costs calculations.
