# Supplementary material for: Impact of difficult-to-treat depression for patients and society: a real-world study
Source: Front Psychiatry. 2025 Dec 9;16:1702137. doi: 10.3389/fpsyt.2025.1702137 (PMC12724535; doi:10.3389/fpsyt.2025.1702137)
Supplement: Supplementary file 2 [file Table2.docx]

**Supplemental eTable 2. Clinical characteristics of emergency-first patients according to the depression profile and complications.^a^**

| Clinical characteristics | Major Depressive Disorder  (n= 224) | Major Depressive Disorder with Suicide Risk  (n= 202) | Difficult to Treat Depression  (n=10) | Difficult to Treat Depression with Suicide Risk  (n=54) | Significance  (p-value)^b^ |
| --- | --- | --- | --- | --- | --- |
| **First CGI-S measure** |  |  |  |  | <0.001 |
| Not evaluated | 60 (26.8%) | 12 (5.9%) | 1 (10%) | 4 (7.4%) |  |
| Borderline mentally ill | 1 (0.4%) | 0 (0%) | 2 (20%) | 1 (1.9%) |  |
| Mildly ill | 55 (24.6%) | 35 (17.3%) | 3 (30%) | 5 (9.3%) |  |
| Moderately ill | 85 (37.9%) | 112 (55.4%) | 2 (20%) | 32 (59.3%) |  |
| Markedly/ Severely ill | 23 (10.3%) | 43 (21.3%) | 2 (20%) | 12 (22.2%) |  |
| **Treatment outcomes** |  |  |  |  |  |
| Inadequate administrative follow-up | 73 (33%) | 89 (44%) | 5 (50%) | 33 (61%) | 0.001 |
| Non-response to treatment^c^ | 73 (53.7%) | 98 (61.8%) | 7 (77.8%) | 41 (85.4%) | 0.001 |
| Probable relapse of mental disorder | 32 (14%) | 48 (24%) | 2 (20%) | 26 (48%) | <0.001 |
| > 75% attendance to Psychiatry consultation | 93 (41.5%) | 104 (51.5%) | 9 (90%) | 36 (66.75%) | 0 .014 |
| > 75% attendance to Psychology consultation | 14 (6.3%) | 41 (20.3%) | 4 (40%) | 19 (35.2%) | 0.098 |
| **Healthcare resource use** |  |  |  |  |  |
| Non-psychiatric Hospitalization | 103 (46%) | 121 (59.9%) | 6 (60%) | 42 (77.8%) | <0.001 |
| Non-psychiatric Emergency consultation | 224 (100%) | 201 (99.5%) | 10 (100%) | 54 (100%) | n.s |
| Psychiatric Hospitalization | 95 (42.4%) | 113 (55.9%) | 6 (60%) | 39 (72.2%) | <0.001 |
| Psychiatric Emergency consultation | 224 (100%) | 202 (100%) | 10 (100%) | 54 (100%) | n.s |
| **Most used antidepressant treatment strategy during the follow-up** |  |  |  |  | <0.001 |
| Psychotherapy and other non-antidepressant drug | 6 (2.8%) | 3 (1.5%) | 0 (0%) | 0 (0%) |  |
| Antidepressant monotherapy | 59 (27.3%) | 57 (28.6%) | 0 (0%) | 1 (1.9%) |  |
| Combination of antidepressants | 46 (21.3%) | 42 (21.1%) | 0 (0%) | 3 (5.6%) |  |
| Augmentation (antipsychotic or mood stabilizer) | 105(48.6%) | 97 (48.7%) | 10 (100%) | 50 (92.6%) |  |
| Missing data | 8 (3.5%) | 3 (1.4%) | 0 (0%) | 0 (0%) |  |
| Mean number of antidepressants | 1.8 (1.1) | 1.8 (1.2) | 3.9 (1.9) | 3.0 (1.2) | <0.001 |
| **Yearly direct medical and indirect mean costs (€/patient/year)^d^** |  |  |  |  |  |
| **Psychiatric direct medical costs** | 3800.4 [7732.0] | 4178.2 [6488.5] | 5561.2 [6555.4] | 8010.6 [9058.7] | .002 |
| Use of mental healthcare resources | 3406.8 [7381.4] | 3618.4 [6057.9] | 4677.1 [6018.4] | 6518.6 [8469.2] | <.001 |
| Psychiatric medications | 393.6 [725.3] | 559.8 [881.1] | 884.1 [834.4] | 1492.0 [1466.0] | <.001 |
| **Somatic direct medical costs** | 1192.6 [2220.8] | 1228.3 [1866.9] | 1856.2 [1159.2] | 2252.8 [3008.0] | .009 |
| Use of general healthcare resources | 1147.1 [2208.6] | 1155.6 [1802.1] | 1399.7 [1105.2] | 1750.3 [2956.9] | .023 |
| Medications for somatic disorders | 45.5 [149.8] | 72.7 [241.3] | 456.5 [737.3] | 502.5 [718.2] | <.001 |
| **Global costs** | 5894.1 [9811.3] | 6708.1 [10135.9] | 11429.2 [12710.1] | 15358.1 [16415.1] | <.001 |
| Use of mental and general healthcare resources | 4553.9 [7624.6] | 4774.0 [6707.4] | 6076.8 [6757.4] | 8268.9 [8873.0] | <.001 |
| Psychiatric and somatic medications | 439.1 [763.3] | 632.5 [968.1] | 1340.6 [1092.3] | 1994.5 [1843.7] | <.001 |
| Temporary work disability | 351.2 [2798.4] | 656.5 [3622.7] | 4011.8 [8496.9] | 2146.3 [6837.4] | <.001 |
| Permanent work disability | 549.9 [3684.9] | 645.1 [4096.2] | 0 [0] | 2948.4 [8493.0] | .005 |

^a^Data are means [Standard Deviation], or number (%).

^b^The characteristics of the patients who contacted first the emergency setting were compared according to the depression profile.

^c^The percentage of non-responders has been calculated over the N of patients with available CGI measurement.

^d^Use of mental healthcare resources: psychiatric admissions, psychiatric emergencies, psychiatric consultation and psychotherapy, neurostimulation and neuromodulation treatments; psychiatric medications: antidepressant, anxiolytics and antipsychotics; use of general healthcare resources: non-psychiatric admissions, non-psychiatric emergencies, non-psychiatric consultations, lab studies and imaging for somatic disorders. Indirect costs due to temporary and permanent work disability were included in global costs calculations.
